# Rural-Urban Differences in the Prevalence of Chronic Pain Among Adult Cancer Survivors

**DOI:** 10.1001/jamanetworkopen.2025.49972

**Published:** 2025-12-17

**Authors:** Hyojin Choi, Emily Hallgren, Maija Reblin, J. Kathleen Tracy, Erika Ziller

**Affiliations:** 1 Department of Family Medicine, The Robert Larner MD College of Medicine, University of Vermont, Burlington; 2 Department of Medicine, The Robert Larner MD College of Medicine, University of Vermont, Burlington

## Abstract

This cross-sectional study uses data from the National Health Interview Survey to examine differences in chronic pain among rural and urban cancer survivors.

## Introduction

Chronic pain prevalence among cancer survivors is double that of the general US population^1^ and associated with many negative outcomes.^2^ Rural residence is often associated with debilitating long-term survivorship effects,^3^ and rural adults experience chronic pain at higher overall rates than urban adults.^4^ However, data are lacking on whether this chronic pain disparity exists for rural cancer survivors, a gap we address by examining rural-urban chronic pain differences among cancer survivors using national survey data.

## Methods

In this cross-sectional study, we pooled data from the 2019-2021 and 2023 National Health Interview Survey (NHIS), a cross-sectional survey conducted by the National Center for Health Statistics (NCHS). The NHIS obtains informed consent from participants. The University of Vermont institutional review board determined this study to be nonhuman participants research, and we followed the STROBE reporting guideline. Because we were interested in chronic pain prevalence among recent cancer survivors, we restricted the sample to adults whose latest cancer diagnosis was within the previous 5 years.

Following NCHS conventions, we defined chronic pain as pain on most or all days over the past 3 months. Rural-urban residence was based on the modified version of the NCHS Urban-Rural Classification Scheme for Counties available in the public use NHIS. We categorized adults living in noncore or nonmetropolitan counties as rural residents and all others as urban residents. Covariates included factors associated with chronic pain in prior research: sex, sexual orientation, race and ethnicity (self-reported; Table 1), age, educational level, marital status, income as a percentage of the federal poverty level (FPL), and number of additional health conditions.

**Table 1.** Characteristics of Recent Cancer Survivors, by Rural and Urban Residence^a^

Characteristic	Weighted % or mean (95% CI)			*P* value
	Total	Rural	Urban	
Unweighted No.	5542	1028	4514	NA
Sex				
Male (n = 2591)	48.4 (46.8-50.0)	48.6 (44.9-52.3)	48.4 (46.5-50.1)	.90
Female (n = 2954)	51.6 (50.0-53.2)	51.4 (47.7-55.1)	51.6 (49.9-53.5)	
Sexual orientation				
Lesbian, gay, or bisexual (n = 164)	3.1 (2.5-3.7)	2.4 (1.2-3.7)	3.3 (2.6-3.9)	.30
Heterosexual (n = 5381)	96.9 (96.3-97.5)	97.6 (96.3-98.8)	96.7 (96.1-97.4)	
Race and ethnicity^b^				
Black, Indigenous, or other racial or ethnic minority group (n = 807)	17.2 (15.6-18.8)	11.1 (8.0-14.1)	18.6 (16.8-20.4)	<.001
Non-Hispanic White (n = 4738)	82.8 (81.2-84.4)	88.9 (85.8-92.0)	81.4 (79.6-83.2)	
Age, y				
18-34 (n = 166)	4.1 (3.4-4.8)	3.7 (2.3-5.1)	4.2 (3.4-5.0)	.44
35-64 (n = 1894)	40.5 (38.9-42.2)	38.6 (34.5-42.8)	40.9 (39.1-42.8)	
≥65 (n = 3486)	55.4 (53.7-57.0)	57.6 (53.6-61.7)	54.8 (53.6-61.7)	
Educational level				
<4-y Degree (n = 3384)	65.5 (63.9-67.2)	81.3 (78.7-84.0)	62.0 (60.2-63.9)	<.001
≥Bachelor’s degree (n = 2161)	34.5 (32.8-36.1)	18.7 (16.0-21.3)	38.0 (36.1-39.8)	
Marital status				
Married (n = 2854)	63.5 (62.0-65.1)	65.3 (61.4-69.1)	63.2 (61.5-64.8)	.03
Not married (n = 2691)	36.5 (34.9-38.0)	34.7 (30.9-38.6)	36.9 (35.2-38.5)	
Income as a % of FPL				
<100 (n = 447)	7.9 (6.9-8.9)	12.0 (9.3-14.8)	6.9 (5.9-8.0)	<.001
100-199 (n = 972)	17.3 (16.0-18.6)	25.7 (22.2-29.2)	15.4 (14.0-16.8)	
200-399 (n = 1678)	29.8 (28.4-31.2)	34.4 (30.7-38.2)	28.8 (27.2-30.3)	
≥400 (n = 2448)	45.0 (43.3-46.7)	27.8 (24.1-31.6)	48.8 (47.0-50.7)	
No. of health conditions	1.84 (1.8-1.9)	2.00 (1.9-2.1)	1.80 (1.7-1.9)	<.001
Pain				
Yes (n = 1981)	35.3 (33.7-36.9)	43.0 (39.0-47.0)	33.5 (31.9-35.2)	<.001
No (n = 3564)	64.7 (63.1-66.3)	57.0 (53.0-61.0)	66.5 (64.8-68.1)	

Abbreviations: FPL, federal poverty level; NA, not applicable.

^a^ Data from the 2019-2021 and 2023 National Health Interview Survey. Total unweighted, N = 5545; total weighted, N = 10 016 966; rural-urban combined weighted numbers do not equal the total weighted number due to rounding.

^b^ The National Health Interview Survey includes the following racial and ethnic categories based on respondent self-report: Hispanic, non-Hispanic American Indian or Alaska Native and any other group, non-Hispanic American Indian or Alaska Native only, non-Hispanic Asian only, non-Hispanic Black or African American only, non-Hispanic White only, and other single and multiple races (not specified). Because of small sample sizes, we aggregated these race and ethnicity categories into a dichotomous measure of Black, Indigenous, or other racial or ethnic minority group or Hispanic compared with non-Hispanic White.

Rural-urban differences in the characteristics of cancer survivors and unadjusted prevalence of chronic pain were compared by using χ^2^ and *t* tests (2-sided). We used logistic regression to compare chronic pain prevalence differences between rural and urban cancer survivors, controlling for covariates. We used SUDAAN, version 11 for analyzing correlated data and NHIS multiple imputation files for family income, with results combined across 10 imputations following Rubin rules. All differences were significant at *P* < .05.

## Results

Of 5542 survivors, 51.6% were female and 48.4% were male, and 55.4% were aged 65 years or older (Table 1). Rural survivors were more likely than urban survivors to be non-Hispanic White, have less than a 4-year college degree, have income below 200% FPL, and to have slightly more chronic health conditions on average. At the bivariate level, the prevalence of chronic pain was 43.0% among rural cancer survivors and 33.5% among urban survivors.

Table 2 presents the association between rurality and chronic pain among cancer survivors, controlling for characteristics associated with chronic pain in prior research. These adjustments attenuated but did not eliminate the rural-urban differences in chronic pain we observed in the bivariate prevalence estimates. The model found that the odds of experiencing chronic pain among rural cancer survivors remained significantly higher than among urban cancer survivors (odds ratio, 1.21; 95% CI, 1.01-1.45).

**Table 2.** Logistic Regression Model of the Odds of Experiencing Chronic Pain^a^

Characteristic	OR (95% CI)	*P* value
Rurality		
Urban	1.00 [Reference]	.04
Rural	1.21 (1.01-1.45)	
Sex		
Female	1.00 [Reference]	.17
Male	0.91 (0.79-1.04)	
Sexual orientation		
Heterosexual	1.00 [Reference]	.46
Lesbian, gay, or bisexual	1.16 (0.78-1.73)	
Race and ethnicity^b^		
Black, Indigenous, or other racial or ethnic minority group	0.78 (0.64-0.95)	.02
White	1.00 [Reference]	
Age, y		
18-34	1.00 [Reference]	NA
35-64	1.54 (0.99-2.40)	.06
≥65	1.25 (0.80-1.97)	.33
Educational level		
≥Bachelor’s degree	1.00 [Reference]	<.001
<4-y Degree	1.53 (1.31-1.79)	
Marital status		
Married	1.00 [Reference]	.12
Not married	1.12 (0.97-1.30)	
Income as % of FPL		
<100	2.07 (1.54-2.77)	<.001
100-199	1.48 (1.20-1.83)	.003
200-399	1.30 (1.09-1.55)	.004
≥400	1.00 [Reference]	NA
No. of health conditions	1.32 (1.26-1.39)	<.001
−2 Log likelihood	443.15	NA
Cox & Snell *R*^2^	0.077	NA

Abbreviations: FPL, federal poverty level; NA, not applicable; OR, odds ratio.

^a^ Data from the 2019-2021 and 2023 National Health Interview Survey. Total unweighted, N = 5545; total weighted, N = 10 016 966; rural-urban combined weighted numbers do not equal the total weighted number due to rounding.

^b^ The National Health Interview Survey includes the following ethnic and racial categories based on respondent self-report: Hispanic, non-Hispanic American Indian or Alaska Native and any other group, non-Hispanic American Indian or Alaska Native only, non-Hispanic Asian only, non-Hispanic Black or African American only, non-Hispanic White only, and other single and multiple races (not specified). Because of small sample sizes, we aggregated these race and ethnicity categories into a dichotomous measure of Black, Indigenous, or other racial or ethnic minority group or Hispanic compared with non-Hispanic White.

## Discussion

Our multivariable findings showed that chronic pain was more prevalent among rural than urban cancer survivors even after controlling for covariates, suggesting an association between chronic pain and additional factors. For example, survivorship resources are generally less available in rural areas,^3^ and rural residents may lack access to pain specialists or face insurance challenges accessing pain care.^5^ Policymakers and health systems should work to close this gap by increasing the availability of pain management resources for rural cancer survivors. Approaches could include innovative payment models for integrative medicine in rural areas or supporting rural clinician access to pain specialists.^4,6^ Study limitations include the cross-sectional design and limited information on individual respondents’ use of multimodal pain treatment options. Further research should examine how best to meet the needs of rural cancer survivors experiencing chronic pain.

## Notes

Supplement.
Data Sharing Statement
