# Supplementary material for: Rural-Urban Differences in the Prevalence of Chronic Pain Among Adult Cancer Survivors
Source: JAMA Netw Open. 2025 Dec 17;8(12):e2549972. doi: 10.1001/jamanetworkopen.2025.49972 (PMC12712726; doi:10.1001/jamanetworkopen.2025.49972)
Supplement: Supplement. — Data Sharing Statement [file jamanetwopen-e2549972-s001.pdf]

Data Sharing Statement

Choi. Rural-Urban Differences in the Prevalence of Chronic Pain Among Adult Cancer Survivors. *JAMA Netw Open*. Published December 17, 2025.
doi:10.1001/jamanetworkopen.2025.49972

Data
Data available: No

Additional Information
Explanation for why data not available: The data are public use data owned by the National Center for Health Statistics and readily available to the public through them.
